# Application of Nanomicelles in Enhancing Bioavailability and Biological Efficacy of Bioactive Nutrients

**DOI:** 10.3390/polym14163278

**Published:** 2022-08-11

**Authors:** Lei Li, Yun Zeng, Minyi Chen, Gang Liu

**Affiliations:** 1 State Key Laboratory of Molecular Vaccinology and Molecular Diagnostics & Center for Molecular Imaging and Translational Medicine, School of Public Health, Xiamen University, Xiamen 361102, China; 2 Department of Pharmacy, Xiamen Medical College, Xiamen 361023, China

**Keywords:** bioactive nutrients, nanomaterials, nanomicelles, delivery systems, bioavailability

## Abstract

Nutraceuticals provide many biological benefits besides their basic nutritional value. However, their biological efficacies are often limited by poor absorption and low bioavailability. Nanomaterials have received much attention as potential delivery systems of nutrients and phytonutrients for multiple applications. Nanomicelles are nanosized colloidal structures with a hydrophobic core and hydrophilic shell. Due to their unique characteristics, they have shown great perspectives in food and nutraceutical science. In this review, we discussed the unique properties of nanomicelles. We also emphasized the latest advances on the design of different nanomicelles for efficient delivery and improved bioavailability of various nutrients. The role of nanomicelles in the efficacy improvement of bioactive components from nutraceutical and health foods has been included. Importantly, the safety concerns on nano-processed food products were highlighted.

## 1. Introduction

Nutraceuticals have recently received ample attention due to their health benefits beyond the nutritive role. The majority of nutraceuticals responsible for the positive effects on well-being are bioactive compounds derived from the plant and animal sources. It has been demonstrated that food-derived bioactive compounds could positively affect the major body systems and reduce the risks of chronic diseases [1]. For example, polyphenols from fruits and vegetables are secondary metabolites in plants. They are not essential nutrients but could potentially promote human health [2]. Some lipophilic bioactive compounds such as carotenoids, long chain polyunsaturated fatty acids may also have beneficial effects on health [3,4]. Many studies have suggested that food-derived bioactive compounds exert various biological actions including antioxidant, anti-inflammatory, anticancer, anti-atherosclerotic and antimicrobial activities [5,6,7]. These compounds in the form of isolated molecules or extracts become promising ingredients for functional foods, which have found wide applications in food industries.

**Figure 1:**
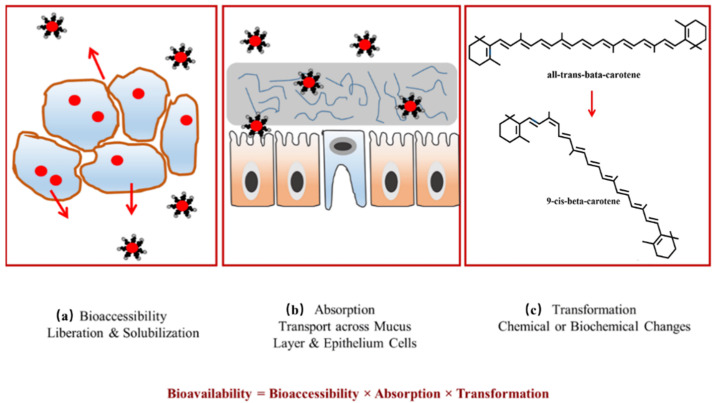
The overall oral bioavailability of bioactives is governed by three main factors: (**a**) bioaccessibility; (**b**) absorption and (**c**) transformation. Reproduced with permission from ref. [9]. Copyright 2016 by MDPI.

In order to exert their health-promoting effects, bioactive compounds need to go through several processes before reaching the target organs, including food processing, being released from the food matrix, and transiting through the gastrointestinal tract and then metabolism [8]. These processes are referred to as bioaccessibility and bioavailability, which are the main factors affecting the biofunctional properties of bioactive compounds [9]. Thus, assessment of bioaccessibility and bioavailability of these compounds has currently become a promising research area. The similar terms have different meanings. Bioaccessibility is the fraction of a compound released from the food matrix, which can be potentially absorbed in the small intestine (Figure 1a). It is the first step of bioavailability [10,11]. Bioavailability is defined as the extent and rate of bioactive compounds absorbed and metabolized by the human body to exert nutritional efficacy (Figure 1b,c) [12]. It is influenced by various factors, including the nature of bioactive compounds such as stability, solubility and composition, the structure of the food matrix and metabolism in enterocytes [13,14,15,16]. Most of plant bioactive ingredients easily degrade and their stability is affected by light, temperature, oxygen and storage. Low aqueous solubility of hydrophobic compounds may result in poor dissolution, limited diffusion and permeability across intestinal epithelium cells, thus affecting their bioavailability [17]. The bioavailability of bioactive compounds could also be modified in the gastrointestinal tract by co-ingested macronutrients and micronutrients presented in the food matrix. These nutrients can interact with bioactive compounds, favoring or inhibiting their liberation and solubilization during digestion [18,19].

High bioavailability is essential for bioactive compounds to reach sufficient blood concentration and exert their beneficial health effects. There are several strategies to be used to enhance their bioaccessibility and bioavailability. They include technological and chemical modifications of the molecules, dosing formulations, combination with other dietary components for synergism, and use of micro-/nanoparticle delivery systems [20]. Recently, a wide range of engineered nanomaterials have been proposed to enhance the nutritional value of food products [21,22,23]. The nanomaterials can be employed to incorporate bioactive compounds to prevent degradation and metabolic modifications [24,25]. Their further benefits include the enhancement of bioavailability, controlled release, effective delivery to specific sites-of-action, masking effect on undesired senses and prevention of interactions with other antagonistic components, etc. [26,27]. Numerous different nanomaterials have been developed for food-related applications, including nanocapsules, nanofibers or nanotubes as delivery systems for bioactive compounds [28]. Nanomicelles are nanosized colloidal dispersions with a hydrophobic core and hydrophilic shell, which have emerged as a promising tool for the delivery of nutrients [29]. This current review provides an overview of the unique properties of nanomicelles endowing them with various functions. The latest advances regarding the design of different nanomicelles for the efficient delivery and improved bioavailability of various nutrients are critically discussed. The role of nanomicelles in the efficacy improvement of bioactive components has also been included. Moreover, the safety concerns on nano-processed food products are also highlighted.

## 2. Types of Nanomicelles

**Figure 2:**
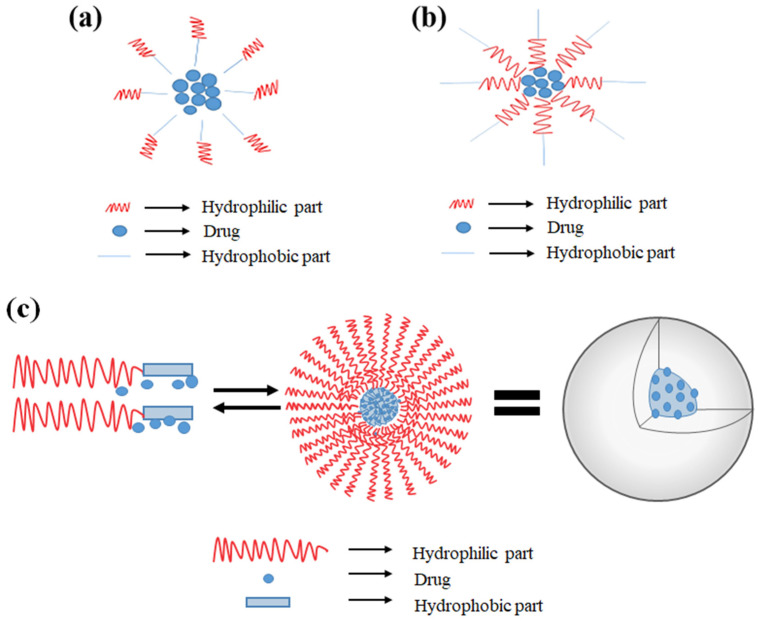
Schematic representation of supramolecular structure of nanomicelles (**a**) regular nanomicelles, (**b**) reverse nanomicelles, (**c**) polymeric micelles. Reproduced with permission from ref. [32]. Copyright 2021 by Wiley.

Nanomicelles usually consist of both hydrophilic (polar) and hydrophobic (nonpolar) groups and amphiphilic molecules are their common subunits [30]. The orientation of these molecules changes to form regular or reverse nanomicelles depending on the solvent. In aqueous medium, the regular nanomicelles are formed with the hydrophilic portion of molecules toward the outer surface and hydrophobic parts inside (Figure 2a) [31]. Conversely, the reverse nanomicelles are formed in the nonpolar solvent with the hydrophobic portion toward the surface and hydrophilic portion toward the core (Figure 2b) [32]. This unique property makes the nanomicelles to be the potential vehicle for loading different types of bioactive compounds. Regular nanomicelles could be used to load nonsoluble compounds and reverse nanomicelles for soluble compounds [33] since the protective shell of nanomicelles could reduce the direct contact of loading compounds with the environment and improve their bioavailability [34,35]. 

Another category is polymeric micelles (PM) with narrow size distributions in the range of 10–100 nm. PM have characteristic core–shell structures including an “inner core” and an “outer shell”, which are usually formed through the self-assembly of block copolymers (Figure 2c). The inner core consists of a hydrophobic core for compound entrapment and the outer shell is composed of a hydrophilic block of polyethylene glycol (PEG), which could protect entrapped compounds from unexpected interactions and biodegradation in an in vivo environment [36,37]. With the unique molecular architectures, PM have high stability and could deliver bioactive compounds to the target site more efficiently. Furthermore, engineering the micelle-forming block copolymers makes it possible for PM to incorporate drug or bioactive compounds on demand, and endow them smart functionalities such as environment-sensitivity and targetability as well. 

## 3. Properties of Nanomicelles

The predominant physico-chemical characteristic of micelles is their small size, which determines their fate in vivo. PM are usually in a diameter range from 10 nm to 100 nm with a substantial narrow distribution. They can evade scavenging by the mononuclear phagocytic system in the liver and bypass the filtration of inter-endothelial cells in the spleen. It is ideal for intravenous injection to attain stable, long-term circulation in the bloodstream [38]. Moreover, PM are typically formed with the hydrophobic parts of the polymer on the inside (core) and hydrophilic on the outside (shell). The hydrophobic core can incorporate drugs with poor aqueous solubility and the hydrophilic shell provides some protection for the drugs against metabolism [39], which leads to improved accumulation of delivered drugs at target tissue sites. All these characteristics are critical in the application of nanomicelles as drug carriers. 

High structural stability is one of unique properties of nanomicelles due to the entanglement of polymer chains in the inner core, and has the two aspects of thermodynamic and kinetic stability [39]. Thermodynamic stability occurs when the concentration of monomers (non-assembled amphiphilic polymer molecule) is above the critical micelle concentration (CMC) [40]. CMC is a threshold for monomers to start to assemble into PM, which is usually affected by the hydrophilic-lipophilic balance of the monomers [41,42]. Nanomicelles generally have very low CMC ranging from 10^–6^ to 10^–7^ M [32]. When above CMC, the polymer chains associate and the self-assemblies/micelles are formed; otherwise, amphiphilic molecules exist separately in aqueous environment. Kinetic stability plays a much more important role in drug delivery in physiological environments at non-equilibrium conditions. When monomer concentration is below CMC, the kinetic stability comes into action, and then nanomicelles disassemble slowly. The slow dissociation could make the nanomicelles remain intact with the delivered drug until it reaches the target site [43]. 

Nanomicelles show excellent ability to load a large number of hydrophobic drugs. Many newly developed drugs are hydrophobic and water insoluble. It is estimated that about 70% of new chemical entities are poorly water soluble, and many are even insoluble in organic media [44]. Low solubility limits the drug dissolution rate and results in erratic absorption patterns. It becomes a key factor to limit the therapeutic efficiency of many potent drugs [45]. With the unique structure, nanomicellles become a promising platform to deliver these poorly soluble compounds. The micelles’ inner hydrophobic core could entrap hydrophobic drug molecules and a hydrophilic outer shell layer extends outwards to maintain the water solubility. They could also prevent biodegradation of entrapped drugs [46,47], leading to a better drug accumulation in the target site. Nanomicelles have been employed as vehicles to deliver chemotherapeutics for cancer treatment. Polyethylene glycol (PEG)-derivatized dual-functional nanomicelles have been established in the clinic to deliver small-molecule drugs such as camptothecin (CPT), doxorubicin (DOX), paclitaxel (PTX), and docetaxel [48,49]. Some other natural products have also been conjugated to PEG to form dual-functional nanomicelles for cancer therapy such as vitamin E succinate, (−)-epigallocatechin-3-gallate, embelin, and S-trans, trans-farnesyl thiosalicylic acid [50,51]. 

Advanced nanotechnology has endowed nanomicelles some novel properties, such as stimuli sensitivity. An ideal micellar system is supposed to deliver the drug, retain its stability during circulation and release it in the target tissue as a result of physiological or external triggers [52]. These nanomicelles are referred to as “stimuli-sensitive nanomicelles”. A variety of triggers have been used to destabilize drug-loaded PM such as temperature, pH, enzymatic reactions, redox processes, light, ultrasound, as well as combinations thereof [53,54]. The pH-responsive PMs are a newly emerged drug nanocarrier for cancer treatment. Generally, block copolymeric micelles containing L-histidine, pyridine, and tertiary amine groups are pH sensitive. When pH is above pKa of protonable group, the copolymers assemble into micelles and the pH-sensitive block forms the core of micelles, which are uncharged and hydrophobic. While pH is below the pKa value, the acidic core is protonated and becomes negatively charged. The ionisation of the polymers leads to increased hydrophilicity and electrostatic repulsion, which in turn causes the destabilisation of the micelles. Therefore, the delivered therapeutic agents could be released selectively in acidic circumstances like a tumor cell, endosomes, or lysosomes [55,56]. Thermo-sensitivity is another property of nanomicelles that is most investigated, especially in the field of oncology. In temperature-responsive systems, drug release is controlled by variation of microenvironmental temperature. The delivery carriers retain the drug load within normal body temperature (~37 °C) and release the drug at higher temperature, for example, at the local temperature of the tumor environment (~40–42 °C) [57]. Thermo-responsive polymers have lower critical solution temperature (LCST), which is a threshold for thermo-responsive polymers to undergo a phase transition [58]. Desired LCST range could be obtained by the introduction of hydrophobic or hydrophilic comonomers [59]. When the temperature is below the LCST, the polymers with thermo-responsive blocks form water-soluble nanomicelles. Whereas, when the temperature is over LCST, hydrogen bonds between water and the polymer chains disrupt and the polymers become insoluble in water and then the destabilization results in the release of delivered drugs. Poly(N-iso-propylacrylamide) (PNIPAAm) and poly(N-alkylacrylamide) compounds have been studied as temperature-responsive PM [54]. Light, including UV, visible, and infrared/NIR light has also been explored as an external trigger for PM to achieve on-demand drug release [60,61]. The light-responsive PM usually are composed of chromophores, such as azobenzene, pyrene, or nitrobenzyl groups [62,63]. When exposed to light, the nanostructure of PM can be altered, leading to disintegration of nanomicelles and then release of payloads [64]. Besides the above-mentioned triggers, hydrolysis, ultrasound and redox potential have also been explored and evaluated in many studies for selective delivery of delivered compounds [65,66,67]. 

Nanomicelles can deliver drugs into tumors via passive accumulation. Their characteristic of small size endows them the ability to extravasate into the interstitium of body compartments with leaky vasculature (tumors and infarcts) by the enhanced permeation and retention (EPR) effect [68]. However, there are still some problems associated with rapid drug release from the micelles and challenges with respect to intracellular delivery of the drug [69]. For active targeting, ligands such as antibodies and peptides could be incorporated on the surface of nanomicelles. They can target cells based on relationships with particular targets and conjugation with locally functioning signal protein [70]. The active nanomicelles could be uptaken by a specific diseased or cancerous cell with maximum distribution and minimum side effects.

## 4. Advantages and Disadvantages of Nanomicelles

Nanomicelles have some advantages due to their size and structural composition. They are an amphiphilic molecule with the characteristic core-shell structure. The hydrophobic drugs can bind to the hydrophobic core of nanomicelles, leading to the enhanced solubility of the drugs by several folds [71]. They can also increase the stability of the drugs, protect them against the elimination by the mononuclear phagocyte system and lead to prolonged blood circulation [70]. Some target moieties such as specific surface receptor, transporter protein, or the phage fusion protein, could be conjugated with nanomicelles, endowing them specific targeting capability [72]. By incorporating or conjugating with special assembly units, PM could release the loaded drugs triggered by various extra- and intracellular biological stimuli, resulting in higher selectivity and lower side effects [73]. Nanomicelles are made of hydrophobic and biodegradable polymeric nanoparticles, which could act as the local depot of drugs for continuous supply of therapeutic agents at the targeted diseased site and improve their treatment effect [74]. PM are formed typically in a diameter range from 10 nm to 100 nm. The size range is considered ideal for intravenous drug delivery without the occurrence of embolism.

The major concern of micelles is their stability in blood dream. When injected intravenously, the nanomicelles are diluted, which may shift the equilibrium towards the unimer state, thus leading to their dissociation. The loaded drugs can leak out of the polymer assembly [75]. Therefore, more strategies should be developed to modify the physical-chemical properties of micelles for high stability. Animal study is necessary to gain further insight to micelle stability. It may provide answers to the fundamental questions which cannot be answered using fluorescence techniques.

## 5. Application of Nanomicelles in Bioactive Nutrient Delivery

### 5.1. Enhance Stability and Bioavailability of Delivered Bioactive Nutrients

**Figure 3:**
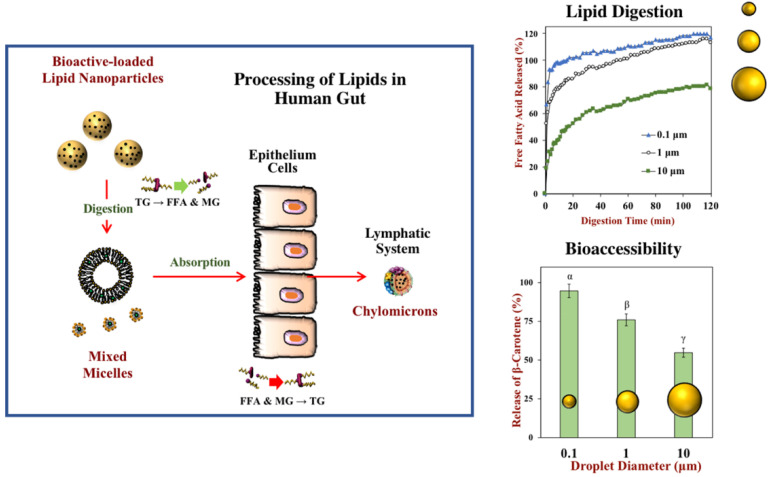
Bioavailability of hydrophobic bioactive substances can be increased by encapsulating them in nanoparticles. The triglycerides (TG) in the lipid nanoparticles are broken down into free fatty acids (FFA) and monoglycerides (MG), which are packed into mixed micelles with bioactive substances and transported to the epithelium cells. They are then reassembled into triglycerides, packed into chylomicrons, and transported into the bloodstream through the lymphatic system. Lipid digestion and nutraceutical bioaccessibility increase with decreasing droplet size. Reproduced with permission from ref. [78]. Copyright 2020 by the American Chemical Society.

Some advantages of nanomicelles contribute to the improved bioavailability of bioactive compounds. Nanomicelles can increase stability during digestion, facilitate transport across the biological barriers, enhance the solubility of poorly water-soluble nutraceuticals, decrease biological and environmental degradation of sensitive compounds, realize target delivery and controlled release. Therefore, they offer a promising solution for the problems regarding solubility, stability and oral bioavailability of bioactive nutraceuticals (Figure 3) [76,77,78], and have been investigated as delivery vehicles for hydrophobic nutraceuticals [28,79]. Some recent applications of nanomicelles in delivering food nutrients and bioactives have been summarized in Table 1.

**Figure 4:**
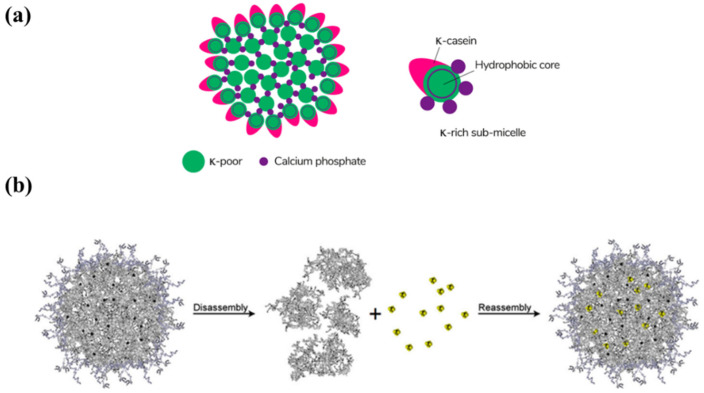
(**a**) The schematic of the submicelle model of the casein micelle. Reproduced with permission from ref. [93]. Copyright 2021 by the MDPI; (**b**) Graphical representation of the general principle behind reassembled casein nanospheres. Briefly, the micellar structure is disrupted, lipophilic compounds dissolved in organic solvents bind to hydrophobic regions of casein peptides; then caseins are reassembled, creating new, substance-loaded nanoparticles. Reproduced with permission from ref. [94]. Copyright 2017 by Springer.

Casein is phosphorylated protein, accounting for about 70~80 percent of total protein in milk. It demonstrates high stability at temperatures >100 °C and pressures up to 100 MPa. Due to its elastic structural and functional properties, casein is regarded as an excellent encapsulation particle and recognized as GRAS (Generally Recognized as Safe). Casein micelles (CM) are one of natural nanovehicles for hydrophobic nutraceuticals. They are in the form of spherical colloidal particles with the average diameter of 150 nm (50–500 nm), consisting of α_s1_-casein, α_s2_-casein, β-casein, and κ-casein [92]. κ-casein is on the surface of CM, providing a hydrophilic, charged, and diffuse surface layer (Figure 4a) [93]. The micelles are stabilized by casein structures and calcium-phosphate bridges. Many studies have tried to produce reassembled (or reformed) casein micelles (r-CM) to encapsulate hydrophobic compounds (Figure 4b) [94].

CM could not only entrap the hydrophobic chemotherapeutic drugs for oral drug delivery, but also provide excellent target-activated release of bioactives in the stomach [95]. Vitamin D (VD) is a fat-soluble vitamin and plays a great role in calcium and phosphorus homeostasis [96]. VD deficiency is a public health problem, and about one billion people suffer from VD deficiency in the world. VD fortification, especially in milk and its products proves to be an efficient way to achieve healthy level of VD. Haham et al. produced r-CM loaded VD_3_ by using ultra-high-pressure homogenization [81]. Their study indicated that r-CM could significantly prevent thermal degradation of encapsulated VD_3_ and keep it stable during cold storage. The high bioavailability of VD_3_ encapsulated in r-CM in vivo was confirmed as well, which was at least as high as VD_3_ in a standard aqueous supplement stabilized with Tween-80. It was also found CM had a strong intrinsic affinity to bind vitamin A and could act as carriers for the fat-soluble vitamin used to fortify commercially available skim milks [83]. Docosahexaenoic acid (DHA) is a kind of Omega-3 polyunsaturated fatty acid, and has lots of beneficial effects on the human body such as cardiovascular-protective and cancer-preventive effects, antithrombotic, antiatherogenic and anti-inflammatory properties [97,98,99]. DHA is hydrophobic and practically insoluble in water, and highly prone to oxidation due to its polyunsaturated structure. The r-CM was introduced to delivery DHA. In the study of Zimet et al., they bound DHA to casein and then entrapped it within re-assembled casein micelles to develop the DHA-loaded r-CM [85]. The system exhibited remarkable protection against DHA oxidation and demonstrated good colloidal stability and bioactive conservation throughout shelf life at 4 °C. CM was also exploited as a carrier of curcumin for cancer therapy. Curcumin is a natural polyphenolic compound extracted from the rhizome of turmeric (Curcuma longa) with multiple bioactivities [100,101]. But curcumin is lipophilic with extremely low solubility in aqueous solution and poor bioavailability [102]. Moreover, it is prone to chemically degrade when exposed to light. Many studies have been conducted to increase its aqueous solubility and bioavailability through encapsulation in various nanoparticles [103,104,105]. Sahu et al. developed the complex formation of curcumin with bovine CMs [87]. The CM-curcumin complex exhibited cytotoxic effects on HeLa cells with IC_50_ of 12.69 µM, which was compared to an equal dose of free curcumin with IC_50_ of 14.85 µM. Because casein is an edible protein, the complex may become a potential oral dose of curcumin for cancer therapy. In addition to antitumor activity, curcumin is a food-grade photosensitizer and exhibits remarkable antimicrobial activity through redox reaction. Micelles with two surfactants of Surfynol 465 and Tween 80 have been used to encapsulate curcumin [106]. The curcumin in all surfactant solutions prepared from Surfynol 465 or Tween 80 showed high stability and good photoinactivation. Furthermore, the micelle-based delivery system promoted adsorption and the generation of reactive oxygen species in the immediate environment of the microbial cell and then enhanced the photoinactivation.

NanoSolve^®^ is a commercial micellar formulation (Lipoid GmbH, Ludwigshafen, Germany) with a key component of purified phospholipids. Purified phospholipids are natural emulsifier derived from soybean extract and could be dispersed in highly concentrated aqueous solutions of polyol or carbohydrate. They are able to solubilize lipids or lipophilic actives for oral application [107] and form transparent emulsions with particle sizes between 30 and 60 nm. Co-enzyme Q10 (CoQ10) and vitamin E are both lipophilic molecules with excellent antioxidant activity. However, their commercial formulations are usually poorly absorbed in the intestine. A human study conducted by Wajda et al. showed the bioavailability of CoQ10 and vitamin E in NanoSolve formulation was increased fivefold and tenfold when compared to their pure substances [82]. In another study, a water soluble micellar formulation of α-tocopherol acetate was developed with a particle size of 50 nm (Aquanova AG, Darmstadt, Germany), which could remarkably increase the α-tocopherol acetate concentration in plasma (Aquanova AG, Darmstadt, Germany) [108].

### 5.2. Improve Bioefficiency of Delivered Nutrients and Bioactives for Disease Therapy

With the development of molecular nutrition, nanomicelles have been regarded as an effective platform to deliver various compounds for health purpose. They have been used as vehicles for many bioactive nutrients in the therapy of some chronic non-communicable diseases. The recent applications of nanomicelles in delivering bioactive nutrients for disease therapy have been summarized in Table 2.

**Figure 5:**
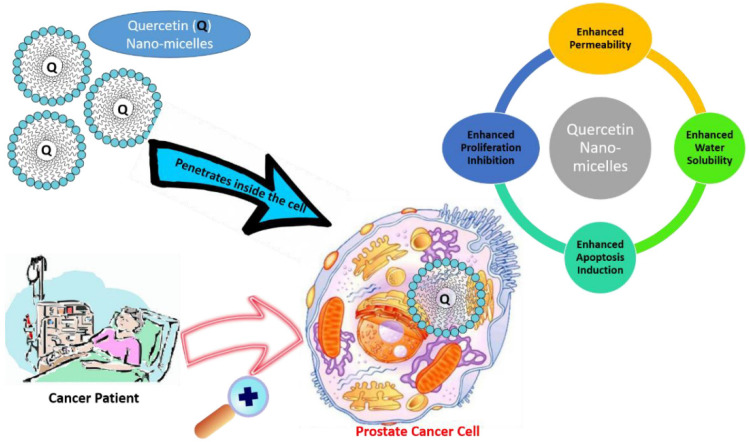
Application of quercetin nanomicelles in providing targeted delivery in prostate cancer therapy. These nanomicelles promote the penetration of quercetin into cancer cells, leading to an increase in bioavailability and subsequent enhancement. Reproduced with permission from ref. [116]. Copyright 2021 by all authors.

Cancer is now widely recognized as a great threat to the health of people. Many works have been done on the micellar delivery system for chemotherapeutic drugs such as PTX and docetaxel [109]. Some nanomicellar drugs have advanced to the market. For example, Genexol-PM is a primary mPEG-PLA polymeric micelle loaded with PTX, which was approved by the FDA in 2007 [110]. In addition to chemotherapeutic drugs, some nutrients and phytochemicals have emerged as effective agents for cancer therapy due to their various effects on diverse molecular signaling pathways and having fewer side effects than conventional treatments [111,112]. Quercetin is a polyphenolic compound and rich in many plants, fruits, and vegetables. It exhibits anticancer activity by inhibiting growth of cancer cells and suppressing tumorigenesis and cancer progression. But its water solubility is as low as 0.17–7.7 µg/mL and only 1% in humans and 17% in rats is bioavailable [113,114,115]. Recently, nanomicelles have been implemented to enhance the solubility and bioavailability of quercetin for better therapeutic use. Zhao et al. encapsulated quercetin in nanomicelles. The water solubility of quercetin was improved by 450-fold. About 25% quercetin was released within the initial 2 h, and then it was followed by slow, sustained release during 48 h monitoring period. The quercetin-loaded micelles inhibited proliferation and apoptosis of human androgen prostate cancer cell lines in vitro with the half-maximal inhibitory concentration (IC50) value of 20.2 μM, much lower than free quercetin (>200 μM). Furthermore, the nanomicelles with quercetin showed higher antitumor efficiency in the PC-3 xenograft mouse model, and proliferation rate decreased by 52.03% compared with the control group. They may become a promising vehicle to deliver bioactives for prostate cancer treatment (Figure 5) [116]. Patra et al. developed optimal mixed polymeric micelle formulation to encapsulate quercetin [90]. Drug loading and encapsulation efficiency of the selected formulation were 9.01 ± 0.11% and 90.07 ± 1.09%, respectively. The solubility and stability of quercetin was significantly improved, and quercetin could be released sustainedly from the mixed micelles. Increased in vitro cytotoxicity was found in breast (MCF-7 and MDAMB-231), ovarian (SKOV-3), and multidrug resistant (NCI/ADR) cancer cells compared to free quercetin. Some other bioactive compounds such as curcumin (Gou, et al.) [117,118], and gambogic acid [91] have also been explored by using mixed PM to enhance their therapeutic potential in cancer.

Osteoporosis is a skeletal disease with low bone mass and a deterioration of the bone microarchitecture, leading to increased bone fragility and risk of fractures. It affects mostly postmenopausal women due to the reduction of estrogen. Resveratrol (3,5,40-trihydroxystilbene; RSV) is a natural nonflavonoid polyphenol with two aromatic rings connected through a methylenic bridge. It is presented in many species of plants such as grapes, cocoa, strawberries, tomatoes, peanuts, hop, cranberries, and sugar cane [119,120,121,122]. Due to structural similarity to estrogen, RSV can bind to estrogen receptors and exhibit beneficial effect in osteoporosis [123]. However, due to its lipophilic nature, RSV has low aqueous solubility. It is extensively metabolized and rapidly eliminated, resulting in poor bioavailability. Nanomicelles have been tested on their ability to encapsulate RSV for improved efficacy. A human study with twelve healthy volunteers showed oral bioavailability of RSV in liquid micellar solubilization was significantly higher than the native powder and no side effects were detected [124]. Nie et al. synthesized RSV loaded mPEG-PLA co-polymeric nanomicelles by using the dialysis membrane technique and investigated their osteoporosis preventive effect in ovariectomized Sprague-Dawley female rats [88]. The synthesized nanomicelles had drug loading and encapsulation efficiency of 11 ± 2.3% and 72.8 ± 2.4%, respectively. RSV in the formulation was sustainedly released over a long period of time and its bioavailability was significantly increased. It could not only increase bone mineral density and bone strength, but also facilitate restoration of bone turnover markers of osteocalcin and C-terminal teleopeptide of type 1 collagen, therefore exhibiting prominent protective effects against osteoporosis.

Obesity is a major public health challenge, which can increase the risk of a multitude of diseases and early mortality in people. Effective, safe, and widely available anti-obesity treatments are urgently warranted. Capsaicin is an alkaloid in various species of chili peppers responsible for the pungent sensation in chili peppers [125]. Accumulating evidence has indicated that capsaicin has excellent anti-obesity activity by facilitating fat oxidation and energy expenditure, improving insulin sensitivity, and promoting the white adipose browning in both rodents and adult humans [126,127,128]. However, there are several disadvantages of capsaicin limiting its application as oral supplements, including poor water solubility, low bioavailability, and obvious irritation of the mouth and gastrointestinal tract [129]. Bao et al. successfully developed a nanomedicine by using α-lactalbumin (α-lac) nanomicelles to encapsulate capsaicin and then delivered it directly to adipose tissues by a microneedle technology [89]. In 3T3-L1 adipocyte model, the nanomicelles could regulate adipogenesis and improve mitochondrial biogenesis, leading to reduced lipid droplet content. Dramatic weight loss and adipose tissue browning was also detected in the obese mice model. The naomicelles could activate energy metabolism, increase mitochondrial biogenesis, and induce adipocyte browning markers. Their effect outperformed direct subcutaneous injection of free capsaicin. Other bioactive compounds have also exhibited protective effect against obesity. Xanthohumol is a prenylated chalcone of the female inflorescences (hop cones) [130]. It could decrease adipogenesis, and improve lipid and glucose metabolism in murine models of hyperlipidemia, obesity and type 2 diabetes mellitus [131,132,133], which are the main components of the metabolic syndrome. However, its therapeutic use was limited by the poor oral bioavailability. A micellar solubilization of xanthohumol was applied by oral gavage at a daily dose of 2.5 mg/kg body weight in a preclinical mouse model of diet-induced obesity, diabetes and non-alcoholic fatty liver disease. Plasma xanthohumol was detected at a concentration of 100–330 nmol/L in the mouse model. It could significantly inhibit body weight-gain and glucose intolerance of the mice induced by western-type diet. The micellar solubilization was proved to be an effective way to enhance the bioavailability and beneficial effects of xanthohumol on different components of the metabolic syndrome [134].

**Figure 6:**
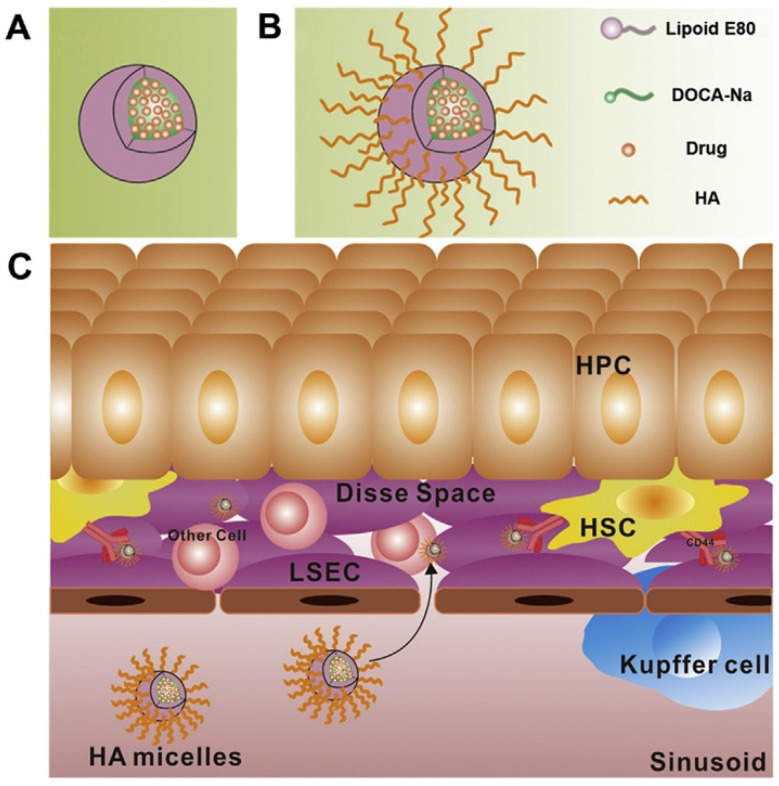
(**A**) Phospholipid bile salt micelles and (**B**) HA micelles fabrication. (**C**) Strategic illustration for the application of HA micelles to target HSCs. Reproduced with permission from ref. [139]. Copyright 2020 by Chinese Pharmaceutical Association and Institute of Materia Medica, Chinese Academy of Medical Sciences. Production and hosting by Elsevier B.V.

Hepatic fibrosis is one kind of chronic liver diseases with a high incidence and mortality in the world. Its most fundamental pathogenesis is the activation and proliferation of hepatic stellate cells (HSCs) [135]. Inhibiting the activation of HSCs becomes an important way for the treatment of hepatic fibrosis. Although there are some drugs, such as silibinin and interferon have been proved to be effective in reversing the activation of HSCs [136]. However, it is still difficult for these drugs to reach HSCs and exhibit their anti-hepatic fibrosis effect in vivo due to the physiological structure of liver. Hyaluronic acid (HA) is a kind of polysaccharides consisting of alternating units of N-acetyl-D-glucosamine and glucuronic acid [137]. It has been demonstrated that HA could specifically bind to CD44 receptors which are usually overexpressed in fibrotic liver [138]. HA has been employed to fabricate HA-functionalized nanomicelles (HA micelles) for target delivery of anti-hepatic fibrosis drugs. Li et al. designed silibinin-loaded hyaluronic acid (SLB-HA) micelles and intravenously injected into rats (Figure 6) [139]. The pharmacokinetic profile in vivo showed the release of SLB-HA continued for 12 h and the blood circulation time was greatly extended. The area under curve was 30.4-fold higher than that of SLB solution. The SLB-HA micelles showed significant liver-targeting effects and could selectively kill activated HSCs. Moreover, it had a good biological safety and biocompatibility. The novel nanomicelle system proved to be a potential vehicle for anti-hepatic fibrosis drugs delivery.

Nanomicelles have exhibited lots of advantages as a delivery system. Many PM formulations have reached clinical trials and some have obtained regulatory approval or clinical evaluation, such as Genexol^®^ PM, NK105 and so on [140]. These drugs are all for cancer indications. As mentioned above, many nanomicelle formulations delivering food nutrients and bioactives have been reported in the literature. However, most of them are currently in experimental research phase. By now, there is no nanomicelle formulation with food nutrients and bioactives to reach clinical trials.

**Table 2:** Application of nanomicelles in delivering bioactive nutrients for disease therapy.

Disease	Bioactives	Nanomicelle Formulation	Size (nm)	Cell Line or Animal Model	Possible Mechanism	References
Cancer	Quercetin	DSPE-PEG2000	13.21 ± 0.97	Human prostate cancer cell line PC-3; PC-3 xenograft mouse model	Inhibit growth of cancer cells and suppress tumorigenesis and cancer progression	[113]
	Quercetin	Mixed polymeric micelles obtained from Pluronic polymers, P123 and P407	24.83 ± 0.44 (A16); 26.37 ± 2.19 (A22)	SKOV-3 (ovarian), NCI/ADR (multidrug resistant), MCF-7 and MDA-MB-231 (breast) cancer cells		[90]
	Curcumin	Monomethoxy poly(ethylene glycol)-poly(3-caprolactone) (MPEG-PCL) micelles	27.3 ± 1.3	C-26 colon carcinoma cells; C-26 xenograft mouse model	Suppress proliferation of tumor cells, down-regulate transcription factors NF-kappa B, AP-1 and Egr-1; down-regulate growth factor receptors; and inhibit the activity of c-Jun N-terminal kinase, protein tyrosine kinases and protein serine/threonine kinases.	[118]
	Gambogic acid	Poloxamer 407/TPGS mixed micelles	17.4 ± 0.5	Breast cancer MCF-7 cells; multidrug-resistant NCI/ADR-RES cells	Induce apoptosis of tumor cells, depolymerize microtubule, and downregulate telomerase activity	[91]
Osteoporosis	Resveratrol	mPEG-PLA co-polymeric nanomicelles	52.87 ± 3.8	Ovariectomized Sprague-Dawley female rats	Promote osteoblast-mediated bone formation and inhibit osteoclast-stimulated bone resorption via similar mechanisms to genistein	[88]
Obesity	Capsaicin	α-lactalbumin (α-lac) nanomicelles	30.2	3T3-L1 adipocyte model	Promote the white adipose browning and suppress lipogenesis	[89]
	Xanthohumol	Micellar Xantho-Flav-Solubilisate	-	Mouse model of obesity, diabetes and non-alcoholic fatty liver disease	Decrease adipogenesis and improve lipid and glucose metabolism in murine models of hyperlipidemia, obesity and T2DM	[134]
Hepatic fibrosis	Hyaluronic acid	Hyaluronic acid micelles	44.9 ± 2.1	Rat model of liver fibrosis	Hyaluronic acid can specifically bind to CD44 receptors which are overexpressed in the liver when hepatic fibrosis occurs	[139]

### 5.3. Safety Concerns of Nanomicelles in Nutrition and Food Science

Apart from advantages of nanomicelles in the nutrition and food industry, safety issues associated with the nanomicelles cannot be neglected. Although the material is generally regarded as safe, risk may arise in human beings due to their completely different physiochemical properties in nanostates. Their small size may increase the risk of bioaccumulation within organs and tissues. They may come into contact with cells and their internal components, such as the nucleus, mitochondrion, and membrane, causing the occurring of a variety of diseases [141]. Many studies have shown nano-entities may change the intracellular milieu by disrupting some cellular pathways and functional processes [142], and then exhibit unanticipated effects on the overall functionality of the cellular system [143]. Lots of factors have impact on the toxicity of nanoparticles such as their nature, concentration, length of exposure, and individual sensitivity [144]. Their special characteristic of large surface area to volume ratio may also be the premise of their migration into food and the toxic effect on humans after consumption [145].

The application of nanomaterials in nutrition and food science is increasing at a high rate. However, there are no standard regulatory laws regarding their use in food and agri-sector so far. The lack of necessary knowledge and regulations may pose a risk to the environment and human health. Therefore, it is critical to build up effective guidelines and policies for the safe utilization of nanoparticles in the food industry. A regulatory structure is required to regulate any dangers connected to nanofood and the usage of nanotechnologies in the food industry. Economic, social, and ethical issues raised by nanotechnology should also been addressed in the regulations. Public participation in nanotechnology decision making is also necessary to ensure democratic control of these technological advances in nutrition and food area. Moreover, any new nanoparticles must undergo full safety assessments before being used in any food product, and at last it should be in the ingredients list of food products containing any nanoparticles.

## 6. Conclusions and Future Perspective

As nanobiotechnology steps forward, the popularity of nanomaterial in nutrition and the food sector is increasing. Nanomicelles have unique architecture, with all their characteristics incorporated into a single cage. They have lots of advantages such as small size, high stability, stimuli sensitivity, and sustained release for hydrophobic compounds. They could enhance stability and bioavailability of poorly soluble bioactive compounds and offer excellent vehicle systems to deliver these agents to the target tissues. Promising results have been achieved which show that nanomicelles could be applied to personalized therapy of some chronic diseases and thus increase the therapy’s efficiency. However, there are still some issues that need to be addressed. The risk can’t be neglected that nanomaterials utilized as food ingredient or delivery vehicle may cause DNA damage, cell membrane disruption, and cell death, and then affect the overall function of systems. Until now, very few studies in an in vivo environment are available to study the effects of nanofoods on human and animal health. Therefore, the introduction of the nanomaterial into nutrition and food system should be prudent. More research work should be conducted to clarify their possible adverse effects on human being as well as the impact on environment. Appropriate regulations are necessary for the practical application of nanofoods. Compulsory testing, especially safety assessments, should be required. The transparency of these issues and scientific information may alleviate the concerns of consumers and increase the acceptability of nanofoods. The nanomicelles, if managed correctly, may bring great changes in improving bioavailability and bioefficacy of bioactive compounds, which will be beneficial to human health.

## Figures and Tables

**Table 1:** Application of nanomicelles in delivering food nutrients and bioactives.

Category	Compound	Micelles Responsible for the Delivery	References
Vitamins	Vitamin D_2_	Re-assembled casein micelle from micellar casein	[80]
	Vitamin D_3_	Re-assembled casein micelle	[81]
	Vitamin E Vitamin A	NanoSolve^®^ Casein micelle	[82] [83]
Lipids	Fish oil	Casein micelle	[84]
	Vegetable oil	Casein micelle	[84]
	Docosahexaenoic acid (DHA)	Re-assembled casein micelle	[85]
Bioactives	β-carotene	Casein micelle	[86]
	Co-enzyme Q10 (CoQ10)	NanoSolve^®^	[82]
	Curcumin	Casein micelle	[87]
	Resveratrol	mPEG-PLA co-polymeric nanomicelles	[88]
	Capsaicin	α-lactalbumin nanomicelles	[89]
	Quercetin	Polymeric micelles	[90]
	Gambogic acid	Polymeric micelles	[91]

## Data Availability

Not applicable.
